# Costunolide, a Sesquiterpene Lactone, Suppresses Skin Cancer via Induction of Apoptosis and Blockage of Cell Proliferation

**DOI:** 10.3390/ijms22042075

**Published:** 2021-02-19

**Authors:** Sung Ho Lee, Young-Chang Cho, Jae Sung Lim

**Affiliations:** 1 Department of Molecular and Cellular Biology, Baylor College of Medicine, Houston, TX 77030, USA; puzim23@gmail.com; 2 College of Pharmacy, Chonnam National University, Gwangju 61186, Korea; 3 Combinatorial Tumor Immunotherapy Medical Research Center, Chonnam National University Medical School, 264 Seoyang-ro, Hwasun-gun, Jeonnam-do 58128, Korea; 4 Department of Biochemistry, Chonnam National University Medical School, 264 Seoyang-ro, Hwasun-gun, Jeonnam-do 58128, Korea

**Keywords:** costunolide, anti-cancer, proliferation, apoptosis, skin cancer, A431

## Abstract

Costunolide is a naturally occurring sesquiterpene lactone that demonstrates various therapeutic actions such as anti-oxidative, anti-inflammatory, and anti-cancer properties. Costunolide has recently emerged as a potential anti-cancer agent in various types of cancer, including colon, lung, and breast cancer. However, its mode of action in skin cancer remains unclear. To determine the anti-cancer potential of costunolide in skin cancer, human epidermoid carcinoma cell line A431 was treated with costunolide. A lactate dehydrogenase assay showed that costunolide diminished the viability of A431 cells. Apoptotic cells were detected by annexin V/propidium iodide double staining and Terminal deoxynucleotidyl transferase mediated dUTP nick end labeling assay assay, and costunolide induced cell apoptosis via activation of caspase-3 as well as induction of poly-ADP ribose polymerase cleavage in A431 cells. In addition, costunolide elevated the level of the pro-apoptotic protein Bax while lowering the levels of anti-apoptotic proteins, including Bcl-2 and Bcl-xL. To address the inhibitory effect of costunolide on cell proliferation and survival, various signaling pathways, including mitogen-activated protein kinases, signal transducer and activator of transcription 3 (STAT3), nuclear factor κB (NF-κB), and Akt, were investigated. Costunolide activated the p38 and c-Jun N-terminal kinase pathways while suppressing the extracellular signal-regulated kinase (ERK), STAT3, NF-κB, and Akt pathways in A431 cells. Consequently, it was inferred that costunolide suppresses cell proliferation and survival via these signaling pathways. Taken together, our data clearly indicated that costunolide exerts anti-cancer activity in A431 cells by suppressing cell growth via inhibition of proliferation and promotion of apoptosis. Therefore, it may be employed as a potentially tumor-specific candidate in skin cancer treatment.

## 1. Introduction

Non-melanoma skin cancer (NMSC) is the most commonly occurring malignancy, which comprises basal cell carcinoma (BCC) and cutaneous squamous cell carcinoma (cSCC) [1]. BCC, which originates in the basal cell layer of the epidermis, is the most common type of skin cancer, accounting for 80% of skin cancers [2,3]. BCC is not usually life threatening; however, if left untreated, it results in local ulcers, loss of function, and disfiguration of the skin [4]. cSCC, originating in the squamous epidermal layer, is the second most common type of skin cancer, constituting approximately 16% of skin cancers [3]. Unlike BCC, cSCC is significantly more lethal because it tends toward a more aggressive and invasive spread; the ensuing metastasis subsequently leads to morbidity and mortality [5]. Current treatment approaches for NMSC patients are primarily surgical removal and/or radiation therapy, one of which can lead to significant morbidity and other apparent consequences, primarily in the visible area [1]. Therefore, there is a need to develop innovative and cost-effective chemoprevention and treatment strategies while minimizing cosmetic damage as an alternative to conventional NMSC therapy. One of these promising strategies is to identify and develop novel natural health compounds that can specifically target cancer cells with minimal side effects and cosmetic damage.

Researches for the biological effects of phytocomplex could contribute to find out the bioactive single molecule studied [6,7]. Indeed, as previously reported, costunolide-containing phytocomplexes showed cytotoxic properties in various cancer cell lines [8,9,10]. Costunolide is a sesquiterpene lactone first obtained from the costus (*Saussurea lappa* Clarke) root [11] and subsequently isolated from several species of plants, including *Aucklandia lappa* Decne [12], *Laurus nobilis* [13], *Magnolia grandiflora* [14], and *Michelia floribunda* [15]. Sesquiterpenes were known to exhibit anti-cancer properties [16]. Currently, thapsigargin and trilobolide are leading sesquiterpene lactones in anticancer therapy [17,18]. It has been reported that costunolide possesses a variety of biological properties, such as anti-oxidant [19], anti-inflammatory [20], anti-allergic [21], neuroprotective [22], antimicrobial [23], anti-cancer [24], and bone remodeling [25] effects. It has been reported that costunolide exerts anti-cancer effects by inhibiting cell proliferation and by inducing apoptosis in various cancers including colon [24], breast [26], prostate [27,28], liver [29], lung [30], and blood cancer [31].

In this study, selective cytotoxicity of costunolide was measured to clarify whether costunolide exhibit specific anti-cancer effect in the skin. Furthermore, underlying mechanism of action of costunolide on the signaling pathways responsible for cell proliferation and apoptosis was also elucidated.

## 2. Results

### 2.1. Costunolide Reduced Cell Viability in A431 Cells

**Figure 1:**
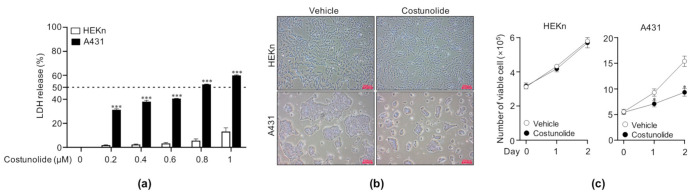
Effect of costunolide on cell viability in HEKn cells and A431 cells. (**a**) Cell viability was measured using the LDH assay kit. Cells were cultured at the indicated concentrations of costunolide for 48 h, following which the supernatants were collected and analyzed. Cells treated with lysis buffer was used as a positive control (100% LDH release). (**b**) Cell growth was detected by microscopic examination. Cells were cultured in the presence/absence of costunolide (0.8 µM) for 48 h. Representative images are shown. Scale bar = 200 µm (**c**) Cell viability was measured using trypan blue exclusion assay. Cells were cultured in the presence/absence of costunolide (0.8 µM) for 48 h. After harvesting and re-suspending, cells were stained with trypan blue solution. The viable cell numbers were counted using a hemocytometer on days 0, 1, and 2. *** *p* < 0.001 as compared to vehicle-treated group.

Prior to gaining insight into the signaling mechanisms involved in the anti-cancer activity of costunolide, we first tested the cytotoxic effect of costunolide on various cancer cell lines (A431, NMSC; B17F10, melanoma; NCI-H460, lung carcinoma; CT26, colon carcinoma) and non-cancerous cell lines (HEKn, skin normal; IMR90, lung normal). We treated the cells with the indicated concentrations (0, 0.2, 0.4, 0.6, 0.8, and 1 µM) for 48 h; thereafter, the supernatants were collected and analyzed using the LDH assay to determine the cytotoxic effect of costunolide. We found that costunolide exerted cytotoxic activity in all cancer cell lines, but it showed cancer-specific cytotoxic activity in the skin only (Figure S1). To investigate the effect of costunolide on cell viability in skin cancer cell line A431, cells were treated with the indicated concentrations of costunolide for 48 h. The supernatants of the cultured cells were assayed to measure lactate dehydrogenase (LDH) release, which is an indicator of the percentage of damaged cells. Human epidermal keratinocytes, neonatal (HEKn) cells were employed as normal control cells. Costunolide exerted significantly reduced cell viability in A431 cells at costunolide concentrations of 0.2, 0.4, 0.6, 0.8, and 1 µM (Figure 1a). The IC_50_ value of costunolide in A431 cells was 0.8 µM. Interestingly, costunolide exerted no significant impact on cell viability in HEKn cells at concentrations below 1 µM. Therefore, subsequent experiments were performed at costunolide concentrations of 0.8 µM. Next, to further confirm the effect of costunolide on cell viability, the cells were cultured in the presence/absence of costunolide for two days. It was revealed that A431 cells treated with costunolide were less confluent than those treated with vehicle at day 2. However, no difference was seen in HEKn cells with/without costunolide treatment (Figure 1b). In addition, trypan blue exclusion assay showed that costunolide significantly reduced the viability of A431 cells (60.43% to vehicle-treated) but not that of HEKn cells (98.06% to vehicle-treated) (Figure 1c). Collectively, costunolide exerted selective anti-cancer activity by inhibiting cell viability.

### 2.2. Costunolide Induced Apoptosis and Suppressed Proliferation in A431 Cells

**Figure 2:**
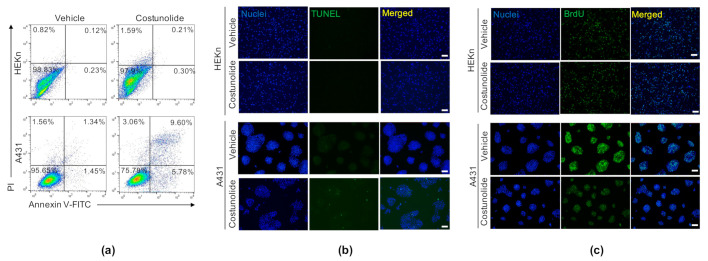
Effect of costunolide on apoptosis and proliferation in HEKn cells and A431 cells. HEKn cells and A431 cells were treated with costunolide (0.8 µM) for 48 h. (**a**) Apoptotic cell death was analyzed by Annexin V/PI staining. Cells were fixed and double stained with Annexin V and PI. Apoptotic cell (Annexin V+:PI- and Annexin V+:PI+) numbers were determined by flow cytometry. (**b**) The fragmented DNA of apoptotic cells was measured using the TUNEL assay. Cells were fixed and double stained with TUNEL and DAPI. Apoptotic cells were determined as green-stained cells under a fluorescence microscope. Scale bar = 100 µm. (**c**) Cell proliferation was determined by the BrdU incorporation assay. After 48 h costunolide treatment, cells were further incubated in BrdU containing culture medium for 2 h. Proliferating cells were identified as green-stained cells under a fluorescence microscope. Scale bar = 100 µm.

Next, we investigated whether the reduced cell viability was due to the induction of apoptosis. To test this, HEKn cells and A431 cells were treated with/without costunolide for 48 h, followed by annexin V/propidium iodide (PI) double staining. As shown in Figure 2a, early apoptotic cells are represented in the lower right quadrant (Annexin V+:PI-) and late apoptotic cells in the upper right quadrant (Annexin V+:PI+). The obtained results showed that costunolide treatment markedly increased the apoptotic cell population among A431 cells (vehicle-treated: 2.79% and costunolide-treated: 15.38%); conversely, this effect was not observed in HEKn cells (vehicle-treated: 0.35% and costunolide-treated: 0.51%). To confirm our finding that costunolide induces late apoptosis in A431 cells, a Terminal deoxynucleotidyl transferase mediated dUTP nick end labeling assay (TUNEL) assay was performed to examine the effects of costunolide on DNA fragmentation, a hallmark of late apoptosis that commits cells to death [32]. Costunolide dramatically induced DNA fragmentation in A431 cells. However, there was no change in DNA fragmentation in HEKn cells following costunolide treatment (Figure 2b). Furthermore, we investigated the effects of costunolide on cell proliferation in HEKn cells and A431 cells using a bromodeoxyuridine (BrdU) incorporation assay. BrdU is a thymidine analog that is readily incorporated into DNA during the S phase of the cell cycle and is a widely used and well-established reagent to label and quantify proliferating cells both in vitro and in vivo [33]. While costunolide did not inhibit the number of BrdU-incorporated cells in HEKn cells, it inhibited both the number of positive cells and the intensity of fluorescence in A431 cells (Figure 2c). Taken together, costunolide selectively induces cell death via cell apoptosis, concomitantly suppressing cell proliferation in A431 cells.

### 2.3. Costunolide Regulates the Bcl-2 Family Proteins in A431 Cells

**Figure 3:**
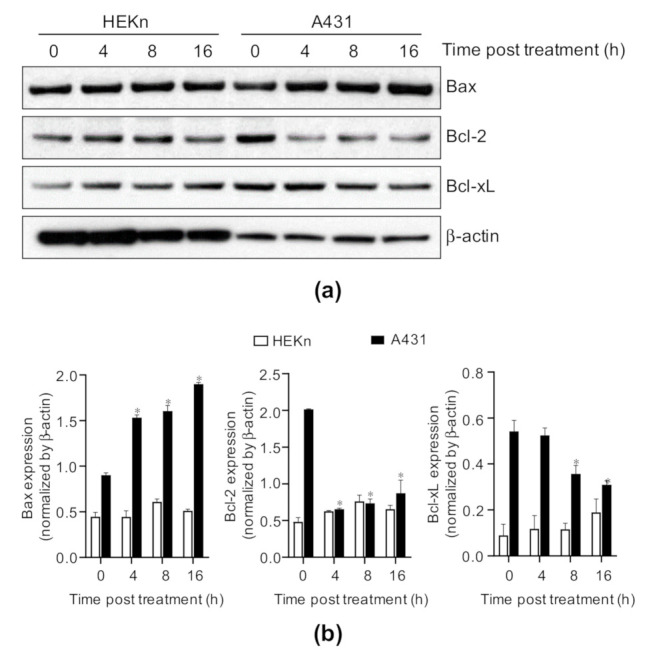
Effect of costunolide on the expression of Bcl-2 family members in HEKn cells and A431 cells. HEKn cells and A431 cells were treated with costunolide (0.8 µM) at various time points (0, 4, 8, and 16 h). Whole cell lysates were harvested. (**a**) Protein levels of the Bcl-2 family (Bax, Bcl-2, and Bcl-xL) members were evaluated by western blotting. β-actin was used as the loading control. (**b**) Bar graphs display quantification of protein levels normalized by β-actin. * *p* < 0.05 as compared to vehicle-treated group.

Given that costunolide induced cell apoptosis, we investigated the effects of costunolide on the Bcl-2 family. HEKn cells and A431 cells were treated with/without costunolide for the indicated time points, and whole cell lysates were analyzed by western blotting to detect the protein levels of Bax, Bcl-2, and Bcl-xL. Costunolide induced an increase in the expression of the proapoptotic protein Bax and a decrease in the expression of anti-apoptotic proteins, including Bcl-2 and Bcl-xL, in a time-dependent manner in A431 cells. However, these changes were not detected in HEKn cells (Figure 3). Therefore, the Bax/Bcl-2 ratio was dramatically increased by costunolide treatment in A431 cells. These results indicated that costunolide selectively induces apoptosis by raising the Bax/Bcl-2 ratio in A431 cells.

### 2.4. Costunolide Triggered Caspase-3 Activation, Poly-ADP Ribose Polymerase (PARP) Cleavage, and Increase in p21 Expression in A431 Cells 

**Figure 4:**
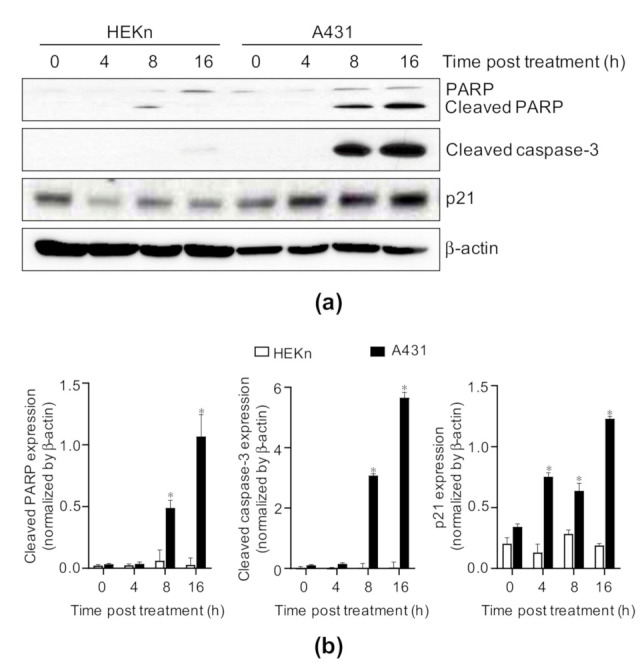
Effect of costunolide on caspase-3 activation and PARP cleavage in HEKn cells and A431 cells. HEKn cells and A431 cells were treated with costunolide (0.8 µM) at various time points (0, 4, 8, and 16 h). Whole cell lysates were harvested. (**a**) Protein levels of PARP, cleaved PARP, cleaved caspase-3, and p21 were evaluated by western blotting. β-actin was used as the loading control. (**b**) Bar graphs display quantification of protein levels normalized by β-actin. * *p* < 0.05 as compared to vehicle-treated group.

Following an increase in the Bax/Bcl-2 ratio in the mitochondrial membrane, mitochondria release cytochrome c into the cytosol, leading to the subsequent activation of caspase-3 for apoptosis [34]. Therefore, the activation of caspase-3 was measured by western blotting to determine the induction of apoptosis. Costunolide significantly induced cleaved caspase-3 (active form) expression (Figure 4). The activation of caspase-3 results in the cleavage of various proteins, the most important one being PARP. PARP is considered to participate in apoptosis, DNA repair and recombination, and the maintenance of chromosomal stability [35]. PARP cleavage leads to its inactivation and subsequently prevents the futile DNA repair cycle [36]. Consistent with the cleaved caspase-3 expression, the cleaved PARP expression was induced in costunolide-treated A431 cells at 8 h post treatment, but it was rarely detectable in costunolide-treated HEKn cells (Figure 4). In addition, the effects of costunolide on the expression of p21, a cyclin-dependent kinase (CDK) inhibitor that regulates the G_1_–S checkpoint, subsequently leading to cell cycle arrest and apoptosis [37], were recorded. Costunolide induced a significant elevation in p21 expression in A431 cells but not in HEKn cells (Figure 4). Taken together, costunolide specifically induced apoptosis in A431 cells through the activation of caspase-3 and PARP, and an increase in p21 expression.

### 2.5. Costunolide Triggered Apoptosis via Activation of JNK and p38 Signaling in A431 Cells

**Figure 5:**
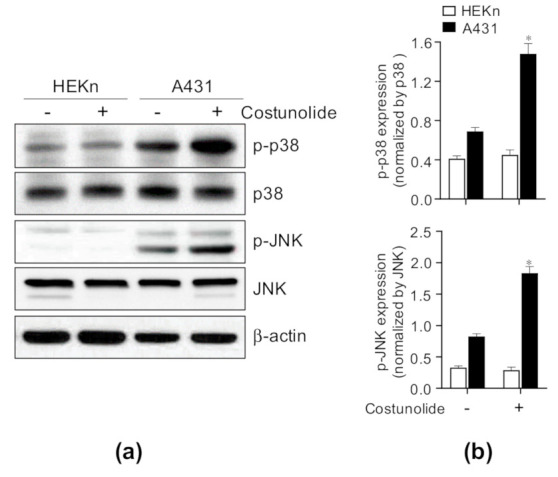
Effect of costunolide on JNK and p38 MAPK Pathway in HEKn cells and A431 cells. HEKn cells and A431 cells were treated with costunolide (0.8 µM) for 4 h. Whole cell lysates were harvested. (**a**) Protein levels of p-p38, p38, p-JNK, and JNK were evaluated by western blotting. β-actin was used as the loading control. (**b**) Bar graphs display quantification of protein levels normalized by β-actin. * *p* < 0.05 as compared to vehicle-treated group.

JNK and p38 MAPK have been known to play a critical role in apoptosis, cell growth, and/or differentiation [38,39]. To investigate the underlying mechanism governing how costunolide induces apoptosis in A431 cells, the cells were treated with/without costunolide for 4 h and whole cell lysates were analyzed using western blotting to determine the activation of JNK and p38. We found that costunolide significantly increased the phosphorylation of JNK and p38 in A431 cells, but not in HEKn cells (Figure 5). These results indicate that costunolide induced cell apoptosis by activating the JNK and p38 pathways.

### 2.6. Costunolide Suppressed Cell Proliferation and Survival via Inhibiting ERK, STAT3, NF-κB, and Akt Activation in A431 Cells

**Figure 6:**
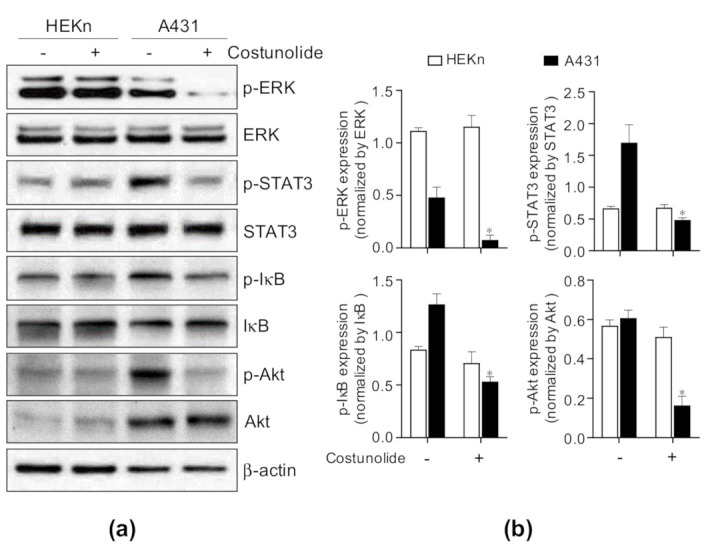
Effect of costunolide on ERK, STAT3, NF-κB, and Akt activation in HEKn cells and A431 cells. HEKn cells and A431 cells were treated with costunolide (0.8 µM) for 4 h. Whole cell lysates were harvested. (**a**) The protein levels of p-ERK, ERK, p-STAT3, STAT3, p-IκB, IκB, p-Akt, and Akt were evaluated by western blotting. β-actin was used as a loading control. (**b**) Bar graphs display quantification of protein levels normalized by β-actin. * *p* < 0.05 as compared to vehicle-treated group.

To determine the molecular mechanism underlying the suppression of cell proliferation by costunolide in A431, the activation of well-established signaling pathways, such as ERK, STAT3, NF-κB, and Akt, was investigated by western blotting. HEKn cells and A431 cells were treated with/without costunolide for 4 h, following which whole cell lysates were analyzed by western blotting. The results showed that costunolide decreased p-ERK expression in A431 cells but not in HEKn cells. Consistent with these results, phosphorylation of STAT3, inhibitor of κB (IκB), and Akt expressions were reduced by costunolide in A431 cells but not in HEKn cells (Figure 6). Collectively, costunolide selectively suppressed cell proliferation in A431 cells via inhibition of ERK, STAT3, NF-κB, and Akt signaling. 

## 3. Discussion

The steady rise in NMSC incidence is likely due to a variety of predisposing risk factors, including inflammation, immune system deficits, nutrient deficiencies, and genetic predisposition toward developing NMSC [3]. The primary treatment for NMSC is either surgical removal or radiation, which can lead to severe cosmetic issues in the skin [1]. Thus, there is an urgent requirement to develop chemotherapeutic and chemopreventive approaches using non-toxic, bioavailable natural agents for NMSC patients. Costunolide is a naturally occurring sesquiterpene lactone that has been studied for its anti-cancer activity in various cancers [40]. However, its impact on skin cancer remains unclear. Thus, we investigated the anti-cancer effects of costunolide in skin cancer and the underlying mechanism of action. 

The observation that costunolide treatment results in increased LDH release in A431 led us to test whether costunolide influences the growth of A431 cells. Microscopic observation (Figure 1b) and viable cell counting showed that costunolide selectively inhibited cell growth in A431 cells (Figure 1c). The inhibition of cell growth was not observed in HEKn cells treated with costunolide. These anti-cancer effects are generally mediated by two major events. (i) induction of apoptosis, and (ii) suppression of cell proliferation [40,41]. To this end, we investigated whether costunolide can induce apoptosis in A431 cells. Apoptosis is involved in the loss of phospholipid asymmetry of the plasma membrane, cell shrinkage, protease activation, and DNA fragmentation [42]. Annexin V/PI double staining proved that costunolide dramatically induced apoptosis of A431 cells by detecting disruption of phospholipid plasma membrane asymmetry. In addition, the TUNEL assay demonstrated that costunolide induced apoptosis of A431 cells by supporting an increased proportion of DNA fragmented cells (Figure 2b). These data clearly indicated that costunolide induces apoptosis of A431 cells to inhibit growth. 

Apoptosis occurs primarily by two pathways. The first pathway is the intrinsic pathway. It has been reported that costunolide induces mitochondria-mediated apoptosis, as demonstrated by the inhibition of Bcl-2, induction of Bax, and subsequent release of cytochrome c in various cancers, such as human prostate cancer [28] and leukemia [43]. Costunolide treatment induced reactive oxidative stress generation, the phosphorylation of JNK and p38 MAPK, the induction of proapoptotic protein Bax, and inhibition of anti-apoptotic proteins Bcl-2 and Bcl-xL [43]. This subsequently caused reduced mitochondrial membrane potential and cytochrome c release [44]. Together with these findings, our data demonstrated that costunolide treatment induced Bax expression and inhibited Bcl-2 and Bcl-xL expression (Figure 3). Furthermore, we noted that JNK and p38 phosphorylation was upregulated by costunolide treatment in A431 cells (Figure 5), implying that these pathways regulate mitochondria-mediated apoptosis. The second pathway is the extrinsic pathway. It has also been reported that costunolide treatment induces extrinsic mechanisms of apoptosis. Costunolide treatment led to the activation of Fas, caspase-8, caspase-3, and PARP cleavage in the human breast cancer cell line MDA-MD-231 [45]. Accordingly, costunolide augmented cleaved caspase-3 (active form) and cleaved PARP levels in A431 cells. Collectively, costunolide induced apoptosis via both the intrinsic and extrinsic pathways.

Next, to address whether costunolide exerts an anti-cancer effect by suppressing cell proliferation, we performed a BrdU incorporation assay. We found that cell proliferation was dramatically suppressed by costunolide treatment in A431 cells (Figure 2c). Consistent with our findings, several prior studies demonstrated that costunolide hinders the proliferation of various cancer cell lines, including colon, breast, and prostate cancer [24,26,27]. A variety of signaling pathways are involved in decreased cell proliferation following costunolide treatment. For example, costunolide suppressed cell proliferation by inhibiting phosphorylation of mammalian target of rapamycin (mTOR) and its downstream kinases p70S6K and 4E-BP1, and inducing the phosphorylation and nuclear localization of p53 in HCT116 cells [24]. In addition, some studies have demonstrated that costunolide inhibited cell proliferation by blocking the G_2_/M phase of the cell cycle and regulates cyclins and CDKs [29,30]. Upregulation of p21 would be expected to promote cell cycle withdrawal by blocking the activity of the cyclin/CDK complexes [46]. We determined that elevated p21 expression induced by costunolide treatment in A431 may be involved in the suppression of cell proliferation via induction of cell cycle arrest (Figure 4). Consistent with our findings, several studies revealed that costunolide treatment induced the G_2_/M phase of cell cycle arrest via elevation of p21 expression [45,47,48]. 

The STAT pathway regulates the transcription of various genes involved in cell proliferation, development, and tumorigenesis [49]. Among the various STAT family members, STAT3 is well known to play an important role in tumorigenesis, cell proliferation, and cell survival in various tissues including colon [50], breast [51], and pancreas [52]. In particular, it has been reported that STAT3 is essential for the skin cancer development [53], and aberrant activation of STAT3 occurs in skin cancer [54]. Thus, genetic or pharmacological approaches to suppress abnormally activated STAT3 are considered as treatments for cancers [55]. As shown in Figure 6, the expression of p-STAT3 in A431 (skin cancer) is highly upregulated compared to HEKn (normal skin cell). This activation may contribute a positive effect on the proliferation and survival of skin cancer cell. Interestingly, costunolide treatment specifically inhibited the expression of p-STAT3 in A431. These data suggest that costunolide shows anti-cancer effect on skin cancer via suppressing STAT3 signaling pathway.

In addition, the ERK signaling pathway regulates cell proliferation and cell differentiation [56]. Inhibition of the ERK pathway could lead to the induction of apoptosis [57]. This explains our finding that inhibited phosphorylation of ERK may be responsible for the suppression of cell proliferation in A431 cells (Figure 6). Furthermore, the Akt signaling pathway is one of the most critical pathways in regulating cell survival [58]. Inhibition of Akt phosphorylation raises the possibility of hampering A431 cell survival (Figure 6). Lastly, NF-κB induces the expression of anti-apoptotic genes, such as the caspase-8 inhibitor FLIP, the inhibitor of apoptosis proteins c-IAP1/2 and XIAP, and members of the Bcl-2 family of apoptosis regulators. Tumor cells may escape from apoptosis via the NF-κB pathway, which has been identified as one of the hallmarks of cancer [59]. As a result, costunolide treatment inhibited the phosphorylation of IκB, which is a key molecule for the activation of NF-κB, demonstrating that costunolide may induce apoptosis via blockage of the NF-κB pathway (Figure 6). Although further studies are needed to understand the exact molecular pathways underlying the action of costunolide, our study delineates a potential anti-cancer effect of costunolide in skin cancer.

According to previously known reports, structure-activity-relationship (SAR) results for sesquiterpene lactone compounds show that the alpha-methylene-gamma-lactone moiety is an important structure for various physiological activities including anti-inflammatory [60], antioxidant [61], anti-microbial [62], and especially anti-cancer activity [63]. In consistent with other sesquiterpene lactones, SAR studies about costunolide have revealed that the alpha-methylene-gamma-lactone moiety is a key structure on the cytotoxic activity showing an anti-cancer effect [64]. It was reported that synthetic derivatives of costunolide resulted in 2–3 times better cytotoxicity for certain cancer cells than costunolide, along with improved safety indicators [65]. Therefore, it is expected that additional SAR studies on derivatives of costunolide will provide better candidates for anti-cancer agents having high cytotoxicity with enhanced safety index. 

Finally, the most significant point of our study is that costunolide exerts anticancer effects against skin cancer at concentrations that were not toxic in normal cells. Since one of the biggest obstacles seen with many existing anticancer treatments is the selective anticancer effect. For example, a potent cytotoxic effect of thapsigargin, one of sesquiterpene lactones was considered as a novel candidate for anti-cancer agent but it hampered due to high toxicity to normal cells [66]. The mechanism for this selective anti-cancer effect was examined only in our results: (1) costunolide selectively causes changes in the Bcl-2 family, the signaling process involved in apoptosis, and caspase-3 activity in only A431 cells not HEKn. (2) costunolide selectively affects the activity of various signaling mediators (JNK/p38 MAPK, ERK, STAT3, Akt, NF-κB) involved in cell proliferation and survival in only A431 cells not HEKn. As a result, the fact that it did not affect HEKn is explained that it showed a selective anticancer effect in both aspects of apoptosis and proliferation. However, the direct target of costunolide was not identified in our study. Therefore, the research on this part should be conducted to clarify the selective anti-cancer effect of costunolide on skin cancer.

## 4. Materials and Methods

### 4.1. Materials

Costunolide (SML0417) was purchased from Sigma-Aldrich (St. Louis, MO, USA). Stock solution (10 mg/mL) of costunolide was prepared with DMSO. It was aliquoted and store at −20 °C until use. All cell culture-related reagents were purchased from GIBCO-BRL (Grand Island, NY, USA). The lactate dehydrogenase (LDH) assay kit (BCT-LDH500) was purchased from BIOMAX (Seoul, Korea). The Annexin V-FITC Apoptosis Detection Kit (APOAF-50TST) was purchased from Sigma-Aldrich. The DeadEnd^TM^ Fluorometric TUNEL System Kit (G3250) was purchased from Promega (Madison, WI, USA). Anti-BrdU antibody (MCA2060GA) was purchased from AbD Serotec (Hercules, CA, USA). Anti-PARP (#9542), anti-cleaved Caspase-3 (#9664), anti-Bax (#2772), anti-Bcl-2 (#4223), anti-Bcl-xL (#2762), anti-p38 (#8690), anti-phospho-p38 (#9211), anti-ERK (#9102), anti-phospho-ERK (#4370), anti-JNK (#9252), anti-phospho-JNK (#9251), anti-STAT3 (#9139), anti-phospho-STAT3 (#9145), anti-IκBα (#4814), anti-phospho-IκBα (#9246), anti-Akt (#4685), and anti-phospho-Akt (#4060) antibodies were purchased from Cell Signaling Technology (Danvers, MA, USA). Anti-p21(sc-397) and anti-β-actin (sc-47778) antibodies were purchased from Santa Cruz Biotechnology (Dallas, TX, USA). 

### 4.2. Cell Culture 

HEKn cells (C-001–5C) were purchased from GIBCO-BRL. The cells were cultured in Medium 154 (M154500) supplemented with human keratinocyte growth supplement (S0015), 10% fetal bovine serum (FBS), and 1% penicillin/streptomycin (P/S) at 37 °C in a 5% CO_2_ incubator. A431 (human skin carcinoma cell line, CRL-1555), B17F10 (skin melanoma cell line, CRL-6475), IMR90 (lung normal cell line, CCL-186), and CT26 (colon cancer cell line, CRL-2638) cells were purchased from ATCC (Manassas, VA, USA) and maintained in Dulbecco’s modified Eagle’s medium supplemented with 10% FBS and 1% P/S at 37 °C in a 5% CO_2_ incubator. In the case of NCI-H460 (lung cancer cell line, HTB-177, ATCC), cells were cultured in RPMI 1640 supplemented with 10% FBS and 1% P/S at 37 °C in a 5% CO_2_ incubator.

### 4.3. Cytotoxicity Assay 

Cytotoxicity was determined using the LDH Assay Kit according to the manufacturer’s protocol. In detail, cells were seeded in triplicate in 6-well plates (~60% confluence at day 0) and then treated with vehicle (0.1% DMSO) or costunolide (0.2, 0.4, 0.6, 0.8, and 1 μM) for 2 days. After treatment with costunolide, the culture supernatants were collected and 10 μL of supernatants was transferred into 96-well plates, followed by incubation with 100 μL of LDH solution at room temperature (RT) in the dark for 30 min. Then, the optical density (OD) was measured at 450 nm using a microplate reader (Molecular Devices Corp., Menlo, CA, USA). To measure cytotoxicity, following condition samples were prepared as well. (1) For background control, OD was measured from the LDH contained in the completed medium. (2) For negative control, OD was measured from vehicle (0.1% DMSO)-treated cells (3) For positive control, OD was measured from lysis solution-treated cells to determine the maximum amount of LDH that can be released from the cells. The following equation was used to calculate the cytotoxicity (%) by costunolide treatment.

Cytotoxicity (%) = [(OD450_experimental control_ − OD450_background control_) − (OD450_negative control_ − OD450_background_ _control_)]/[(OD450_positive control_ − OD450_background_) − (OD450_negative control_ − OD450_background control_)] × 100 (1)

### 4.4. Trypan Blue Exclusion Assay

HEKn cells and A431 cells were seeded in triplicate in 6-well plates (~60% confluence at day 0). Next day, the cells were treated with vehicle (0.1% DMSO) or costunolide (0.8 μM). After 24 h and 48 h, the cells were washed with 1×PBS twice and resuspended by trypsin treatment. Then the cells were centrifuged at 101× *g* for 5 min. After supernatant removal, cell pellet was resuspended in complete medium 1 mL. Aliquoted 10 μL of suspension was mixed with 10 μL of 0.4% trypan blue solution (T8154, Sigma-Aldrich). After 3 min incubation, unstained cells (viable cells) were counted using a hemocytometer within 5 min.

### 4.5. Analysis of Apoptotic Cell Death 

Apoptotic cell death was determined using the Annexin V-FITC Apoptosis Detection Kit according to the manufacturer’s protocol. In detail, HEKn and A431 cells (~60% confluence at day 0) were seeded in 100-mm dish. Next day, the cells were treated with vehicle (0.1% DMSO) or costunolide (0.8 μM). After 48 h, the cells were washed with PBS twice and then detached using a 0.05% Trypsin-EDTA for 5 min. After centrifugation (101× *g* × 5 min), supernatants were removed. Then cell pellet was resuspended with ice-cold PBS. Cell number was counted using a hemocytometer and then diluted as a 1 × 10^6^ cells/mL with 1 × binding buffer. Next, 500 μL of cell suspension was transferred into a new EP tube and Annexin V-FITC (5 μL) and propidium iodide (PI, 10 μL) were mixed and incubated for 10 min at RT in the dark. After staining, flow-cytometry data were acquired using FACSCalibur (BD Biosciences, San Diego, CA, USA) and analyzed using FlowJo software [67] (BD Biosciences). The negative control was vehicle (0.1% DMSO) treated cells.

### 4.6. TUNEL Assay 

The fragmented DNA of apoptotic cells was measured using the DeadEndTM Fluorometeric TUNEL System Kit according to the manufacturer’s protocol. In detail, HEKn cells and A431 cells (~60% confluence at day 0) were seeded on Poly-L-lysine (P8920, Sigma-Aldrich)-coated glass coverslip in 24-well plate. Next day, the cells were treated with vehicle (0.1% DMSO) or costunolide (0.8 μM). After 48 h, the cells were washed with ice-cold 1×PBS twice and then fixed with 4% methanol-free formaldehyde (#15710, Electron Microscopy Sciences, Hatfield, PA, USA) in PBS for 15 min at RT. The fixed cells were washed with 1×PBS twice and followed by permeabilization with 0.2% Triton X-100 (T8787, Sigma-Aldrich) in PBS for 5 min at RT. Next, the cells were washed with PBS twice and incubated with 100 μL of equilibration buffer for 10 min at RT. 50 μL of rTdT incubation buffer was prepared by mixing 45 μL of equilibration buffer, 5 μL of nucleotide mix, and 1 μL of rTdT enzyme per reaction. Sample was incubated for 1 h at 37 °C after adding rTdT incubation buffer. 2×SSC in deionized water was added and further incubate for 15 min at RT and followed by wash with 1×PBS thrice. For nucleic acid staining, 1 μg/mL of DAPI (D9542, Sigma-Aldrich) in PBS was incubated for 20 min at RT in the dark. After wash with 1×PBS thrice, cell-attached glass coverslip was lifted carefully and mount on slide glass with Slow-Fade solution (S36936, Invitrogen, Waltham, MA, USA). Apoptotic cells were identified as green-stained cells under a fluorescence microscope (Nikon ECLIPSE Ts2, Nikon, Tokyo, Japan). The negative control was vehicle-treated cells and positive control was staurosporine-treated cells.

### 4.7. BrdU Incorporation Assay

Cell proliferation was determined by measuring BrdU incorporation into DNA. Cells were treated with vehicle (0.1% DMSO) or costunolide (0.8 μM) for 2 days. After 2 days, the cells were incubated in a culture medium containing BrdU (10 μM) (P5002, Sigma-Aldrich) at 37 °C for 2 h (in case of HEKn cells, incubated for 16 h). The cells were washed with PBS thrice and fixed with 4% paraformaldehyde in PBS for 15 min at RT. Next, the cells were incubated in permeabilization buffer (0.1% Triton X-100 in PBS) at RT for 20 min. After three washes with PBS, the cells were incubated in 1 N HCl on ice for 10 min, and then they were incubated in 2 N HCl at RT for 10 min followed by a 10 min incubation in phosphate/citric acid buffer (pH 7.4) at RT to denature DNA. After three washes with permeabilization buffer, the cells were incubated in antibody staining buffer (0.1% Triton X-100/5% BSA in PBS) containing anti-BrdU antibody at RT overnight and then incubated with anti-rat Alexa Fluor 488 secondary antibody (A11006, Invitrogen) at RT for 1 h. The nuclear DNA of the cells was counterstained with DAPI. Proliferating cells were identified as green-stained cells under a fluorescence microscope.

### 4.8. Western Blot Analysis 

Western blot analysis was conducted as described previously [68]. In detail, cells were incubated with (0.1% DMSO) or costunolide (0.8 μM) for indicated time points. The cells were washed with ice-cold 1×PBS twice and lysed with RIPA buffer (50 mM Tris-HCl pH 8.0, 150 mM NaCl, 0.1% SDS, 0.5% deoxycholate, 1% NP-40, and 1 mM EDTA) supplemented with the protease inhibitor cocktail (P8340; Sigma-Aldrich) and Phosphatase Inhibitor Cocktail I (P5726) and II (P0044) (Sigma-Aldrich) followed by centrifugation (17,532×*g* for 15 min). Supernatants were carefully obtained, and those protein concentrations were measured using a BCA protein assay kit (#23227, Thermo Fisher Scientific, Inc., Waltham, MA, USA) according to the manufacturer’s protocol. Supernatants were denatured in 5 × SDS-PAGE sample buffer (SF2002–110–00, Biosesang, Korea) at 95 °C for 5 min. Denatured protein samples (30 μg) were loaded on 6–12% SDS-PAGE gel (depending on target protein size) and the gel running was performed for 90 min at 100 V. Then protein samples on the gel were transferred to polyvinylidene fluoride membranes for 100 min at 100 V. For blocking, 5% nonfat dry milk (#9999, Cell Signaling Technology) in 1×Tris-buffered saline and Tween-20 (TBS-T; 20 mM Tris-HCl pH 8.0, 150 mM NaCl, 0.1% Tween 20, pH 7.5) was used for 1 h at RT followed by washing with TBS-T thrice. For primary antibody incubation, each antibody was diluted in 5% bovine serum albumin (BSA) as the following dilution factor. Anti-PARP (1:1000), anti-cleaved Caspase-3 (1:700), anti-Bax (1:1000), anti-Bcl-2 (1:1000), anti-Bcl-xL (1:1000), anti-p38 (1:1000), anti-phospho-p38 (1:1000), anti-ERK (1:1500), anti-phospho-ERK (1:1500), anti-JNK (1:1000), anti-phospho-JNK (1:1000), anti-STAT3 (1:1000), anti-phospho-STAT3 (1:1000), anti-IκBα (1:1000), anti-phospho-IκBα (1:1000), anti-Akt (1:1200), anti-phospho-Akt (1:1200), anti-p21(1:1000) and anti-β-actin (1:4000). The membranes were incubated with primary antibody for 16 h at 4 °C followed by washing with TBS-T thrice. For secondary antibody incubation, secondary antibody [anti-mouse IgG-HRP (#7076, Cell Signaling Technology) and anti-rabbit IgG-HRP (sc-2004, Santa Cruz Biotechnology)] was diluted in TBS-T (1:2000). The membrane was incubated with secondary antibody for 1 h at RT followed by washing with TBS-T. SuperSignal West Pico (#34087, Thermo Fisher Scientific, Inc.) and Femto (#34095, Thermo Fisher Scientific, Inc.) reagents were used to detect HRP-conjugated secondary antibodies. Chemiluminescence was detected using a Luminescent Image Analyzer (Amersham ImageQuant800, GE Healthcare, IL, USA). To quantify the protein expression, ImageJ software [69] was used (National Institutes of Health, Bethesda, MD, USA). The expression of phospho-protein was normalized by each total protein but the expression of other proteins was normalized by β-actin. 

### 4.9. Statistical Analysis 

Statistical analysis was performed using GraphPad Prism software [70] (version 5.0; GraphPad, Inc., San Diego, CA, USA). Data are expressed as mean ± standard error of the mean of at least three independent experiments. The differences between experimental groups were analyzed for statistical significance using the non-parametric Mann–Whitney U test and were considered significant at *p* < 0.05. All experiments were performed three or more times with similar results, independently under identical or similar conditions.

## 5. Conclusions

In conclusion, we discovered that costunolide exerts anti-cancer activity in skin cancer. Costunolide inhibited cell growth via induction of apoptosis and suppression of cell proliferation in A431 cells. Notably, these anti-cancer effects were not observed in HEKn cells. Taken together, our data indicated that costunolide possesses immense potential as a safe and potent chemotherapeutic agent against skin cancer in humans.

## Data Availability

The data presented in this study are available in the article or Supplementary Material.

## Supplementary Materials

The following are available online at https://www.mdpi.com/1422-0067/22/4/2075/s1, Figure S1. Effect of costunolide on cell viability in various cancer and non-cancerous cell lines.
