# Supplementary material for: Costunolide, a Sesquiterpene Lactone, Suppresses Skin Cancer via Induction of Apoptosis and Blockage of Cell Proliferation
Source: Int J Mol Sci. 2021 Feb 19;22(4):2075. doi: 10.3390/ijms22042075 (PMC7922093; doi:10.3390/ijms22042075)
Supplement: Supplementary file 1 [file ijms-22-02075-s001.pdf]

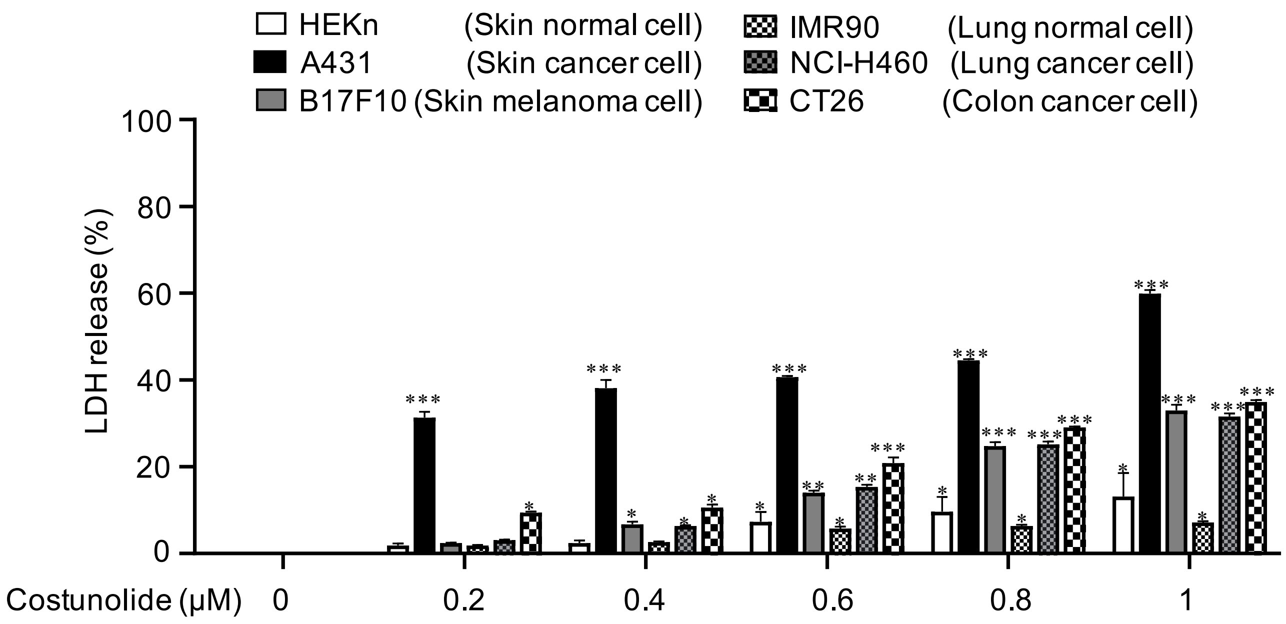

Figure S1. Effect of costunolide on cell viability in various cancer and non-cancerous cell lines. Cell viability was measured using the LDH assay kit. Cells were cultured at the indicated concentrations of costunolide for 48 h, following which the supernatants were collected and analyzed. Cells treated with lysis buffer was used as a positive control (100% LDH release). **p* < 0.05, **p* < 0.01 and ****p* < 0.001 as compared to untreated group.
